# Natural enemy defense, provisioning and oviposition site selection as maternal strategies to enhance offspring survival in a sub-social bug

**DOI:** 10.1371/journal.pone.0195665

**Published:** 2018-04-25

**Authors:** Maurilio López-Ortega, Trevor Williams

**Affiliations:** 1 Instituto de Biotecnología y Ecología Aplicada (INBIOTECA), Universidad Veracruzana, Xalapa, Veracruz, Mexico; 2 Instituto de Ecología AC (INECOL), Xalapa, Veracruz, Mexico; Universidade Federal de Vicosa, BRAZIL

## Abstract

The influence of maternal defense against natural enemies, maternal provisioning and oviposition site selection on offspring survival before and after hatching were examined in a semelparous pentatomid bug, *Ramosiana insignis*. Oviposition occurs on leaves of *Schoepfia schreberi*, or surrounding vegetation from which nymphs migrate to feed exclusively on *S*. *schreberi* flower buds. Oviposition is asynchronous; the mother lays additional eggs immediately prior to hatching of the core brood that rapidly consume the additional eggs. In the absence of maternal defense egg masses were more heavily parasitized, suffered ant predation and an increased prevalence of sibling cannibalism. Maternal provisioning in the form of addition eggs significantly reduced the prevalence of sibling cannibalism of core brood eggs. Migration of the core brood away from the oviposition site was also significantly higher in the absence of maternal provisioning. If not consumed, additional eggs were capable of producing viable progeny of both sexes, indicating that they were in fact marginal progeny. The average clutch size on non-host vegetation was numerically greater than clutches laid on host trees (borderline significant P = 0.058). A greater number of additional eggs were deposited with clutches laid on non-host vegetation compared to those on the host plant. Egg masses on non-host vegetation were less likely to be discovered by parasitoids, compared to those on the host tree. Overall, clutches on non-host vegetation produced one third more offspring than clutches on the host tree. We conclude that *R*. *insignis* females present a remarkable combination of maternal defense, provisioning of additional eggs and oviposition site selection as strategies to enhance offspring survival in both the egg and nymph stages.

## Introduction

The decisions made by insects on where to lay eggs are expected to have a major influence on the survival, growth, and reproductive potential of the offspring, especially for species in which the immature stages have limited dispersal abilities [1, 2]. Apart from abiotic factors, the survival of phytophagous insects will depend on the availability of food resources and the presence of natural enemies [3]. Females in search of oviposition sites may reduce the likelihood of discovery by predators and parasitoids by choosing to oviposit in physical refuges such as inside plant tissues [4, 5], on non-host vegetation [6], on poor quality host plants that are not frequently visited by natural enemies [7–9], or in areas of high plant diversity that reduce the searching efficiency of natural enemies [10, 11].

Semelparous females that lay single batches of eggs can improve offspring survival by investing part of their resources in physical defense of the eggs and early juvenile stages of their progeny from the attack of natural enemies (the maternal defense hypothesis) [12, 13]. Offspring survival may be further improved by parental regurgitations [14], provisioning of food items [15], or the production of trophic eggs, which are non-viable eggs or egg-like structures produced by the female as food for her offspring [16, 17]. Maternal provisioning of nutritional resources may reduce offspring mortality through starvation (the provisioning hypothesis) or reduce the prevalence of sibling cannibalism (the cannibalism reduction hypothesis). The relative importance of each of these functions will depend on the severity of the risk of starvation faced by offspring [18].

The production of trophic eggs has been observed in many taxa, including invertebrates such as snails, spiders and insects [18]. Trophic eggs often have a morphologically or biochemically specialized phenotype to meet the nutritional needs of offspring. Trophic eggs are non-fertile so that nutritional resources are not used by the developing embryo [16].

In many cases the survival of offspring cannot be predicted when food resources vary greatly over time and space. In such cases parents may opt to produce a core brood of high quality individuals and a group of marginal offspring that can be sacrificed if necessary to improve the survival of their siblings [19]. The marginal offspring may be reared to increase parental fitness when nutritional resources are abundant or used to replace members of the core brood that die during rearing [20].

One means by which parents may confer a handicap to marginal offspring is through asynchronous egg production in which the late-hatching offspring are at a clear disadvantage compared to their older and larger siblings. For species with egg-feeding offspring, the production of marginal offspring through asynchronous egg production can also be viewed as a mechanism by which parents provide food resources to the core brood to avoid offspring starvation [21], or reduce the prevalence of sibling cannibalism among the members of the core brood [19, 22]. In a manner analogous to the production of trophic eggs, the importance of the provisioning and cannibalism reduction functions will depend on the risk of starvation during offspring development, and the relative costs of producing core and marginal offspring [18].

In the present study we examined the strategies employed by a semelparous univoltine pentatomid bug, *Ramosiana insignis* (Blanchard) to improve the number and survival of offspring. We demonstrate that these involve a combination of maternal defense of the core brood, asynchronous egg production and oviposition site selection. First, we examined the benefits of physical maternal care on the survival of eggs until eclosion in the presence of natural enemies (the maternal defense hypothesis). Next we examined whether additional eggs were produced to improve nymph survival (provisioning hypothesis) or to reduce sibling cannibalism (cannibalism reduction hypothesis). As the insect lays its eggs on both host and non-host plants, we then examined the consequences of oviposition site selection on maternal provisioning and the risk of succumbing to natural enemy attack. As migration from the non-host to the host plant is likely to be energetically costly and potentially risky in terms of predation, we hypothesized that mothers on non-host plants should invest more heavily in food provisioning to offspring and only use non-host plants for oviposition if non-host plants provided a specific survival advantage to offspring. As these studies indicated that additional eggs were provided to meet the nutritional needs of core offspring, we then asked whether the prevalence of sibling cannibalism and nymphal survival differed on host and non-host plants when an alternative food source (host plant flower buds) was available that may be able to substitute for the nutritional role of additional eggs. Finally, we examined whether core offspring avoided the consumption of additional eggs laid by their mother in favor of eggs from an unrelated female, as a mechanism to avoid cannibalism of marginal siblings.

## Materials and methods

### Life cycle of the insect

**Fig 1:**
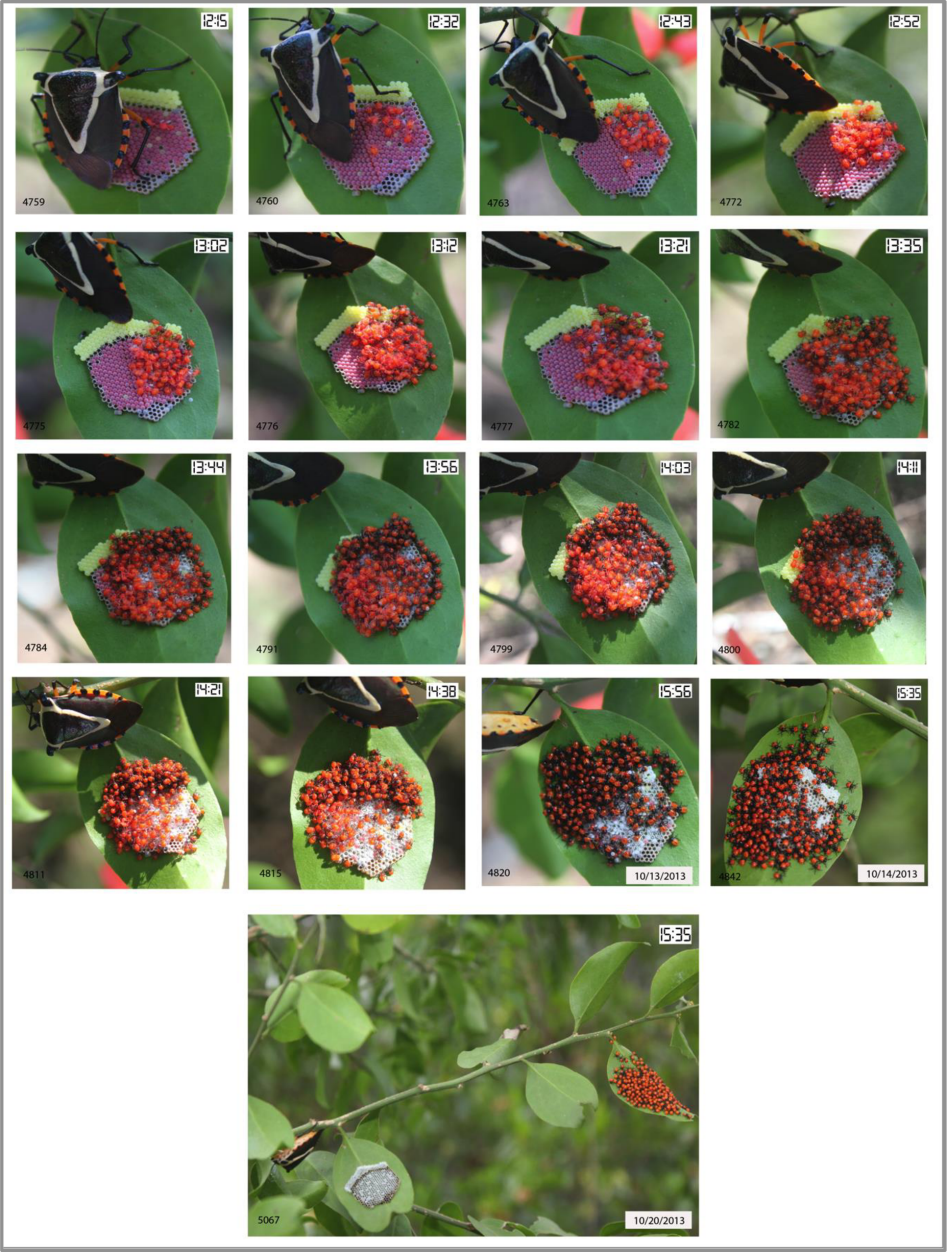
Time series of photographs of eclosion of *Ramosiana insignis* nymphs. Nymphs begin hatching from core brood (pink) eggs at 12.15 hrs on 10/13/2013 and immediately consume additional yellow-colored eggs on underside of a leaf of *Schoepfia schreberi* host plant over the following 2 hrs. Images dated 10/14/2013 and 10/20/2013 show presence of core brood at 1 day and 7 days post-hatching. Date format is mo/day/yr.

The pentatomid bug, *R*. *insignis*, is distributed from Mexico to Panama [23]. The insect feeds exclusively on the flower buds, shoots and developing fruits (drupes) of the Gulf greytwig tree, *Schoepfia schreberi* J.F. Gmel (Olacaceae). In Mexico, eggs are laid in single masses on the underside of *S*. *schreberi* leaves or on the non-host vegetation that surrounds host trees in the period September-October, near the end of the rainy season [24, 25]. We have named this first batch of eggs "core brood". Mothers remain over the egg mass and are often observed kicking away predators and parasitoid wasps. After approximately 3 weeks, eggs turn pink and the female deposits a second batch of yellow-colored eggs at one extreme of the egg mass, usually that closest to the leaf petiole (Fig 1). We call the second batch of eggs "additional" eggs. A few days later the nymphs emerge synchronously and feed on the additional eggs over a period of 2–4 h, during which the cuticle undergoes sclerotization (Fig 1). Following sclerotization the nymphs do not feed again on additional eggs. The nymphs remain closely associated with the natal egg mass and the mother for 12–14 days until they molt to the second instar, whereupon they migrate *en masse* in search of food. The mother remains close to the natal site and dies some days later. For nymphs that emerged on the host plant, migration to the developing flower buds of *S*. *schreberi* normally takes just a few minutes, whereas for those that emerged on non-host vegetation, finding the host plant requires descending to ground level followed by a group migration to the base of the nearest host tree and then the ascension of the tree that, taken in all, can last several days. Feeding and juvenile development continues until the adult stage is reached in March-April which is the end of the dry season. Adult bugs enter an inactive phase until late in the rainy season when copulation takes place and oviposition activity begins once more [24, 25].

### Study site

The study site is privately owned land to which the owners authorized access during the period of the study. The site comprised a triangular area of 1.97 km^2^ defined by the three small villages of Tejerias—altitude 924 m (19° 21' N, 96° 54' W), Monte Blanco—altitude 977 m (19° 22' N, 96° 55' W), and Osto—altitude 838 m (19° 18' N, 96° 50' W), in the state of Veracruz, Mexico. Two additional sites were also sampled in one experiment: Volador—altitude 703 m (19° 21' N, 96° 46' W) and Llano Grande—altitude 870 m (19° 21' N, 96° 53' W), at a distance of 9 km and 3 km from the Tejerias site, respectively. The predominant vegetation in this area is a mixture of animal grazed pasture with fragments of oak forest, associated with coffee plantations, secondary vegetation and low deciduous forest. Observations were performed with reference to individual mature *S*. *schreberi* trees, of 3–5 m height, that are scattered across the pasture land of the study area.

### General methods

The study was performed between 2007 and 2014. Gulf greytwig trees were carefully examined for the presence of *R*. *insignis* and their egg masses during the period September 2007 to March 2008 and September to December 2008. The height of egg masses from the ground was measured and recorded. At the same time, the surrounding vegetation, comprising mainly annual plants, was similarly examined in a 10 m radius around each tree. The height of egg masses from the ground and the distance to the nearest host tree was measured and recorded. The fate of eggs was determined by gently moving the guarding female to one side of the egg mass, photographing each egg mass using a digital camera (Canon EOS Digital Rebel XTi, Canon Inc.), and counting the total number of eggs, and the numbers of parasitized, predated, hatched and unhatched eggs. Females immediately re-adopted their position over the egg mass following photography. Parasitism was determined by the melanized (gray) appearance of parasitized eggs whereas predation was determined by the presence of fragments of traces of egg chorion left by predatory ants (validated in previous observations). Cannibalism was determined by examining core brood eggs that failed to hatch for the presence of a puncture hole and a crumpled appearance of an otherwise intact egg chorion. As other hemipteran predators were never observed feeding on egg masses, we were confident that puncture marks in unhatched eggs were the result of sibling cannibalism. Female bugs guarding their eggs were marked on the dorsal thorax using a spot of non-toxic acrylic paint and egg masses were marked using a small colored piece of plastic tape placed ~30 cm away from the egg mass, for future identification. Females and their egg masses were re-examined at weekly intervals until emergence and dispersal of the progeny. Unless otherwise stated, all mean values are given with the corresponding SE value.

### Benefits of maternal care: The maternal defense hypothesis

A total of 49 *R*. *insignis* females guarding their egg masses were located on S. *schreberi* trees between September 2007 and March 2008. Of these, 26 randomly selected females were removed as soon as oviposition was observed (prior to laying the additional batch of eggs), and 23 females were left guarding their eggs. Females that were taken away from their egg mass never returned to the oviposition site. Observations on the fate of eggs were performed at weekly intervals. The prevalence of parasitism, predation and cannibalism was determined based on those eggs that failed to hatch. The number of progeny nymphs was counted and recorded.

### Effect of asynchronous oviposition on nymphal survival: The provisioning hypothesis

A total of 40 female bugs that were about to lay egg masses on S. *schreberi* trees, or that were in the process of oviposition, were located and marked. When experimental females were observed to have laid the additional batch of eggs, an average of 23.3 ± 0.3 (N = 56) days after the first oviposition, they were randomly assigned to one of two groups. For one group, additional eggs were carefully removed from the egg mass using soft entomological forceps, placed in a glass vial and transported to the laboratory where they were placed into plastic dishes with muslin lids and incubated at 18–20°C until progeny emergence. For the other group, additional eggs were left undisturbed with the egg mass and mother. Nymphs began emerging from egg masses approximately 3 days after the oviposition of additional eggs. The prevalence of parasitism, cannibalism and predation was determined as described above. The duration of the period during which nymphs remained associated with their natal egg masses was estimated by direct observations performed at two-day intervals. Nymphs that emerged from additional eggs in the laboratory were allowed to feed on *S*. *schreberi* fruits developing on twigs placed in cages 20x20x20 cm with muslin walls until they reached the adult stage and could be sexed.

### Does oviposition site selection influence maternal provisioning or exposure to natural enemies?

A total of 93 *R*. *insignis* females guarding egg masses were located on *S*. *schreberi* trees and careful searches of the vegetation surrounding each tree revealed the presence of 218 females guarding egg masses on non-host plants. The numbers of core brood eggs and additional eggs, and the incidence of parasitism and predation, cannibalism and egg eclosion was determined and recorded over the entire course of development of each egg mass until the dispersal of progeny nymphs. The height of all egg masses above the ground and distance to the nearest host tree were measured and recorded. Samples of all non-host plants on which egg masses were observed were taken to the laboratory and identified to species, or failing that genus, by direct comparison with specimens held in the herbarium of the Instituto de Ecología A.C. (INECOL), Xalapa, Mexico. Voucher specimens of plants and insects collected during the study were also deposited in the INECOL herbarium and entomological collections.

### Does oviposition site selection influence the functional role of additional eggs?

To determine the influence of oviposition asynchrony on the survival of nymphs, 60 unhatched egg masses with additional eggs and attendant mothers present on *S*. *schreberi* were located at all five sites (Osto, Monte Blanco, Tejerias, El Volador and Llano Grande) in October 2013. Twigs with egg masses were cut, taken to the Tejerias site and each placed in 2 l capacity plastic containers with the attendant mother. Fifteen egg masses were randomly assigned to each of the following four treatments: (i) egg mass with additional eggs, (ii) egg mass but with additional eggs carefully removed using a small blunt spatula, (iii) egg mass with additional eggs and a twig of *S*. *schreberi* flower buds as food, (iv) egg mass with additional eggs removed but with a twig of *S*. *schreberi* flower buds. The base of the twig with flower buds was placed in a sealed tube of water. Containers were sealed with muslin onto which a moist cotton pad was placed as a water source. Containers were placed on shelves protected from rain and direct sunlight by an open-sided roofed shack at the Tejerias site. After hatching, the mother was removed, the number of living nymphs was counted at weekly intervals and twigs were replaced following each count. The phenology of twigs cut from *S*. *schreberi* trees at the study site changed from flower buds to flowers and young fruits during the 6-wk period of the study. The entire experiment was simultaneously performed using 60 egg masses with additional eggs collected from non-host vegetation at the same sites. These egg masses were treated identically to those from *S*. *schreberi* and were randomly assigned to the same four treatments and monitored for the same period.

#### Do nymphs avoid sibling cannibalism of additional eggs if non-siblings are available?

To compare the feeding response and development of nymphs on siblings (marginal progeny) and non-sibling eggs, 10 egg masses were collected in October 2013 in which the mother had recently deposited the additional batch of eggs. Egg masses on leaves were taken to the laboratory and each placed in a 20 cm diameter Petri dish. Egg masses were manipulated as follows–half of the additional eggs were carefully removed from each egg mass using a small spatula leaving half of the original additional eggs. The removed eggs were replaced by equal proportions of two types of eggs: (i) additional eggs from a different mother and (ii) core brood eggs from a different mother that were in the yellow-pink stage and were several days away from eclosion. Petri dishes containing individual leaves with manipulated egg masses and a moist pad of cotton were placed in the laboratory at 20–23°C. Egg masses were then observed at hourly intervals during daylight and were observed continuously following eclosion of nymphs to detect feeding preferences. Following eclosion these broods were taken to the open-sided shack at the Tijerias site and their development was monitored daily for the following 2 weeks until nymphs had molted to the second instar when they would normally migrate to feed on flower buds in the field.

### Statistical analyses

The influence of female removal, additional egg removal or oviposition site on numbers of eggs and nymphs in each clutch and the heights of clutches above the ground were checked for normality and homogeneity of variances and compared by t-test. The relationship between parasitism and distance to the nearest host tree was examined by Spearman’s rank correlation. Where possible, proportional data were analyzed by fitting generalized linear models (GLMs) with a binomial error distribution specified in GLIM 4 [26]. Where necessary, minor overdispersion was taken into account by scaling the error distribution [27]. The proportion of egg parasitism or predation, proportion of cannibalized eggs and the ratio of additional to core brood eggs were unsuitable for GLM and were subjected to Mann-Whitney U test. Body size (pronotum width) and live weight of females from host and non-host plants were compared by t-test. The frequencies of migrating cohorts in the presence or absence of additional eggs were compared by χ^2^ test. The survival of nymphs given access to additional eggs or host plant flower buds was analyzed by fitting Weibull models in RStudio (v. 1.0.153), the results of which are given as χ^2^ statistics. All statistical procedures, with the exception of fitting generalized linear models and survival analyses, were performed using the Statistica v. 7.1 package (Statsoft Inc., Tulsa, OK).

## Results

### Benefits of maternal care: the maternal defense hypothesis

**Fig 2:**
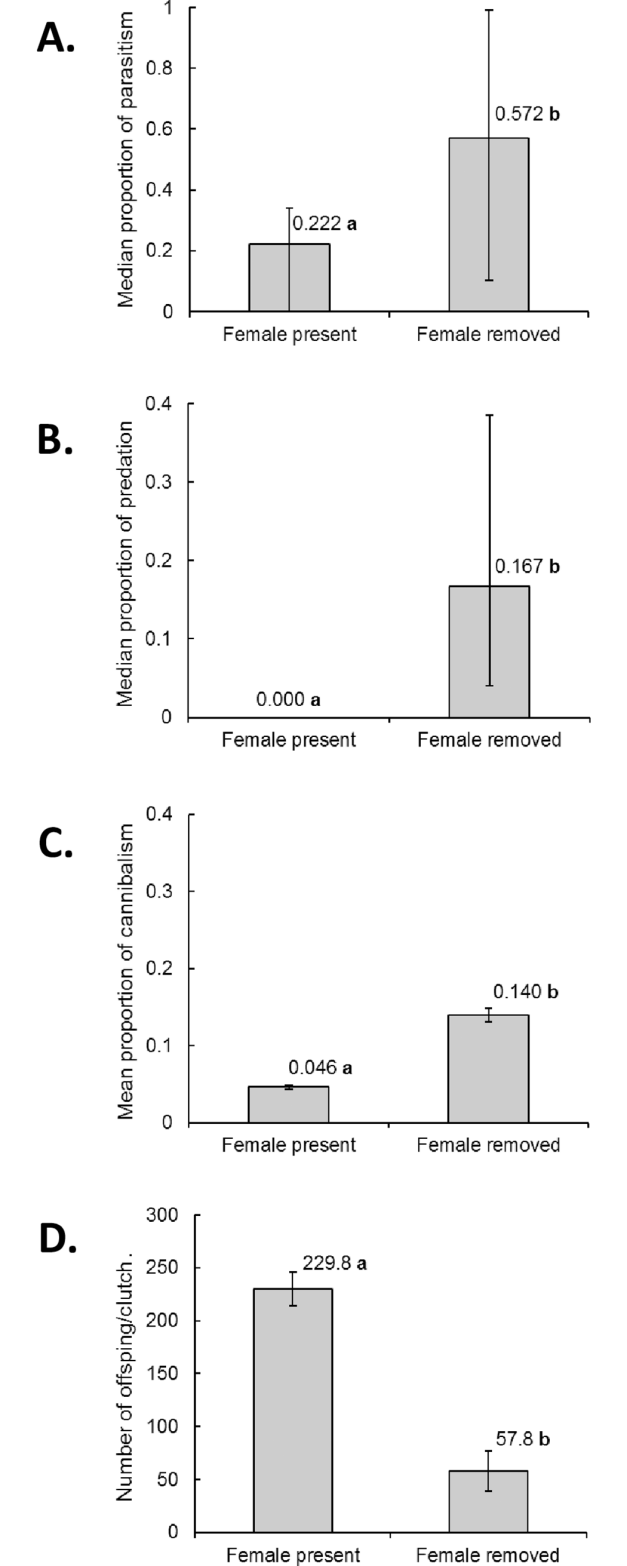
**Effect of female removal on proportion of core brood eggs (A) parasitized, (B) predated, (C) cannibalized and (D) average number of nymphs that emerged in each clutch.** Values above columns indicate median (A, B) or mean (C, D) values. Values followed by identical letters did not differ significantly (P > 0.05). Vertical bars indicate interquartile range for median values and standard error for mean values.

Mean (± SE) clutch size for core brood eggs did not differ significantly between the treatments involving females that remained with their eggs (mean 304.5 ± 7.4, N = 23) and females that were removed (mean 308.3 ± 12.1, N = 26) (t = 0.225; d.f. = 47; P = 0.822). Core brood eggs without maternal care were more heavily parasitized than cared-for eggs (U = 182; P = 0.019) (Fig 2A). Almost no egg predation occurred in the presence of the mother (three eggs were predated from a single clutch), whereas one third of the eggs (mean 105.3 eggs/clutch; median proportion 0.167) were predated in the absence of maternal care (U = 60; P < 0.001) (Fig 2B). The most abundant parasitoid was a species of *Telenomus* (*Telenomus* sp. 2), whereas the most frequent predators were ants (the identity of natural enemies identified during the study is given in S1 File).

The absence of the female (and the additional eggs that she provided) also resulted in a significantly higher prevalence of cannibalism of core brood eggs compared to eggs that were cared for over the entire course of their development (χ^2^ = 156; d.f. = 1; P < 0.001) (Fig 2C). As a result, the mean number of nymphs produced by each female was four-fold higher in clutches defended by the mother compared to clutches lacking maternal defense in which additional eggs were not present (t = 6.87; d.f. = 47; P < 0.001) (Fig 2D).

### Effect of asynchronous oviposition on nymphal survival: The provisioning hypothesis

The mean number of additional eggs that were laid in the egg-removal treatment (78.0 ± 6.3) was similar to that of the not-removed treatment (85.4 ± 9.5) (t = -0.646; d.f. = 38; P = 0.522). Parasitism of core brood eggs was similar when additional eggs were removed or remained with median proportion parasitism values of 0.277 and 0.227, respectively (U = 155; P = 0.223). In contrast, the prevalence of cannibalized eggs was slightly and significantly higher when additional eggs were removed (median proportion 0.023) compared to the non-removal treatment (median proportion 0.006) (U = 66; P < 0.001). This did not significantly influence the total number of offspring that emerged in the presence or absence of additional eggs, which was 224.3 ± 13.1 and 188.5 ± 14.5, respectively (t = -1.822; d.f. = 38; P = 0.076). However, observations made at 28 days post-emergence revealed that the number of clutches of offspring that had migrated away from the oviposition site was 10/20 for clutches in which the additional eggs had been removed compared to 3/20 in the case of offspring with additional eggs present (χ^2^ = 5.58; d.f. = 1; P < 0.018). From these results we conclude that that principal function of the additional eggs is to provide nutritional resources to offspring, in line with the provisioning hypothesis.

A high prevalence of parasitism (mean proportion 0.540, range of SE: 0.530–0.555) was observed in the additional eggs that had been removed from oviposition sites. Moreover, in contrast to the majority of studies on provisioning by sub-social insects, additional eggs were found to be fertile and of those that were not parasitized, a proportion of 0.889 produced viable progeny (N = 17 clutches). Both sexes were observed to emerge from additional eggs; the adult sex ratio was 0.39 male (N = 7 cohorts).

### Does oviposition site selection influence maternal provisioning or exposure to natural enemies?

Eggs masses on non-host vegetation were distributed across 39 plant species from 22 families (S1 File). Egg masses were most commonly found on species of the family Rubiaceae with a total of 99 egg masses distributed over six species, followed in order of abundance by Myrtaceae (29 egg masses), Verbenaceae (17), Compositae (14), Malpighiaceae (10) and 17 additional plant families (with a total of 49 egg masses). The species of Rubiaceae that were most commonly selected for oviposition were *Chiococca alba* (L.) Hitchcock (53 egg masses) and *Randia albonervia* Brand (14 egg masses). Despite intensive field observations over a six-year period (2007–2013) nymphs were never observed feeding on plants other than *S*. *schreberi*.

**Fig 3:**
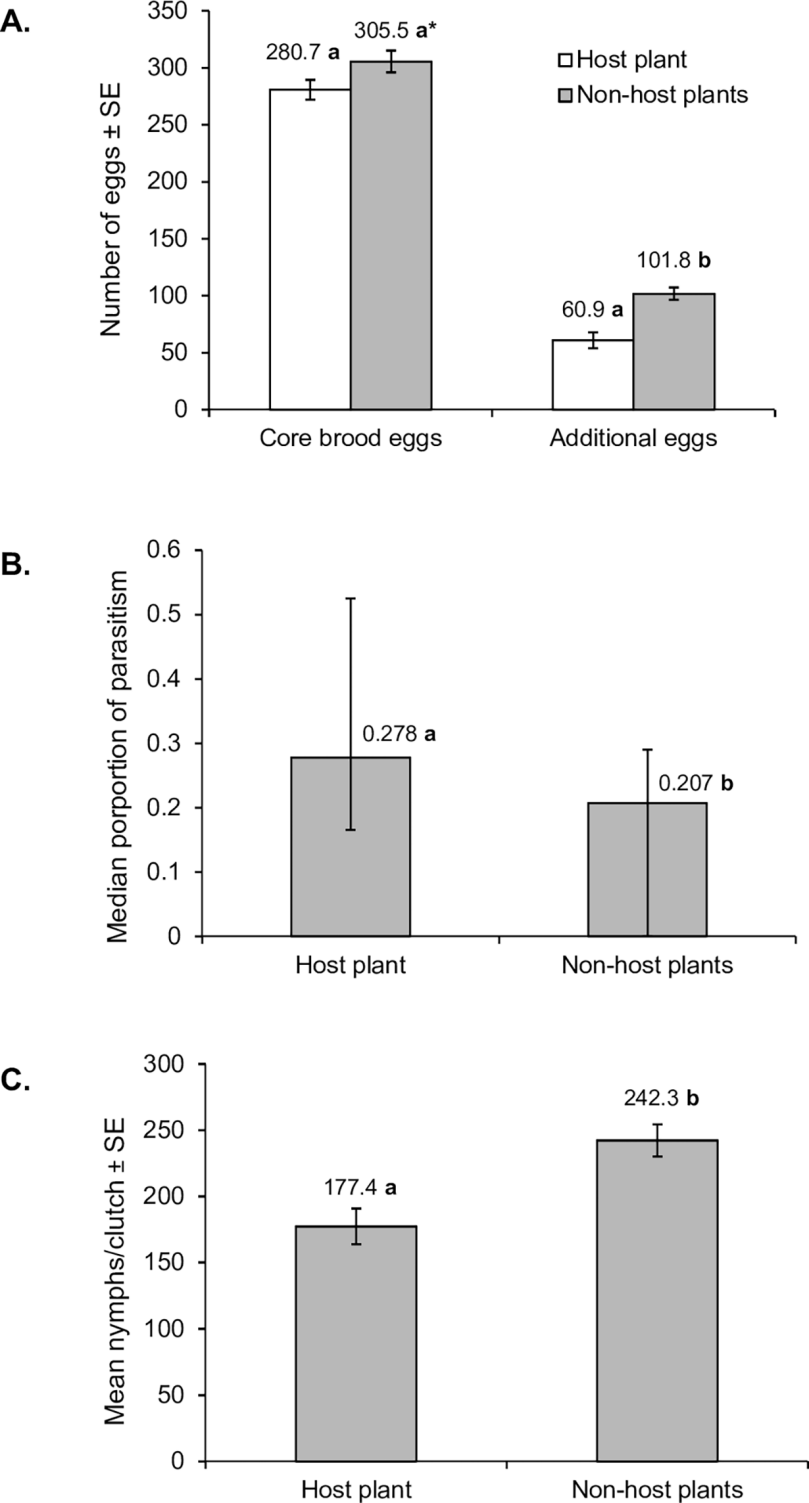
**Effect of oviposition site selection (host tree vs. non-host vegetation) on (A) production of core brood and additional eggs, (B) proportion of core brood eggs parasitized, (C) production of offspring nymphs.** Values above columns indicate mean (A, C) or median (B) values. Values followed by identical letters did not differ significantly (P > 0.05), except for letter marked with asterisk (*) in (A) which was marginally significant (P = 0.058). Vertical bars indicate interquartile range for median values and standard error for mean values.

The average number of core brood eggs laid on non-host vegetation was numerically greater than clutches laid on the host plant, *S*. *schreberi* (Fig 3A), an effect that was borderline significant (t = -1.925; d.f. = 74; P = 0.058). Similarly, a significantly greater number of additional eggs were deposited with clutches laid on non-host vegetation compared to those laid on the host plant (Fig 3A) (t = -4.601; d.f. = 74; P <0.001), such that the ratio of additional eggs to total eggs laid (core brood + additional eggs) was also significantly higher on non-host vegetation (median proportion 0.252) than on the host plant (median proportion 0.173) (U = 323; P <0.001). Body size did not differ significantly between females on host and non-host plants; the mean live body weight prior to oviposition was 881 ± 16 mg (N = 38) for females on host plants compared to 863 ± 20 mg (N = 38) for females on non-host plants (t = 0.679; d.f. = 74; P = 0.498). Similarly, the mean width of the pronotum at its widest point in females from host plants (15.76 ± 0.13 mm, N = 35) and non-host plants (15.86 ± 0.12 mm, N = 36) did not differ significantly (t = 0.538, d.f. = 69, P = 0.592).

Overall, the prevalence of egg parasitism of core brood on non-host vegetation (Fig 3B) was significantly lower than on the host plant (U = 463; P = 0.007). This was because significantly fewer egg masses laid on non-host vegetation were attacked by parasitoids (23/38) compared to those laid on host plants (32/38) (χ^2^ = 5.68, d.f. = 1; P = 0.017). Levels of parasitism of those eggs masses that were discovered by parasitoids did not differ significantly between host (median proportion parasitism 0.385) and non-host plants (median proportion parasitism 0.278) when unparasitized egg masses were excluded from the analysis (U = 280; P = 0.133). Cannibalism of core brood eggs (median proportion 0.013), was similar for clutches laid on both host and non-host vegetation (U = 697; P = 0.795).

Egg masses laid on *S*. *schreberi* were significantly higher above the ground (2.47 ± 0.13 m) than those laid on non-host vegetation (0.94 ± 0.11 m) (t = 8.876; d.f. = 74; P <0.001). However, within each type of habitat, parasitism (excluding unparasitized clutches) was not significantly correlated with egg mass height from the ground for host plants (r^2^ = 0.002, F_1,30_ = 0.072; P = 0.791) or non-host plants (r^2^ = 0.002, F_1,30_ = 0.050; P = 0.825). Similarly, the height above the ground of egg masses that were discovered by parasitoids, or those not discovered, did not differ significantly for those laid on host plants (mean height of parasitized clutches 2.42 ± 0.14 m, unparasitized clutches 2.81 ± 0.39 m; t = -1.127; d.f. = 36; P = 0.267) or those laid on non-host vegetation (mean height of parasitized clutches 0.92 ± 0.15 m, unparasitized clutches 0.99 ± 0.19 m; t = -0.345; d.f. = 36; P = 0.732). The average horizontal distance between egg masses laid on non-host vegetation and the nearest *S*. *schreberi* tree was 3.01 ± 0.15 m (N = 214). There was no significant correlation between distance to the nearest *S*. *schreberi* tree and prevalence of parasitism in egg masses on non-host plants (Spearman's r_s_ = -0.131, P > 0.05).

A greater proportion of core brood eggs laid on non-host vegetation hatched to produce nymphs (median 0.780), compared to those laid on host plants (median 0.699) (U = 456; P = 0.005). Consequently, females that oviposited on non-host vegetation produced one third more nymphs (Fig 3C) than females that oviposited on host plants (t = -3.572; d.f. = 74; P < 0.001).

### Does oviposition site selection influence the functional role of additional eggs?

**Fig 4:**
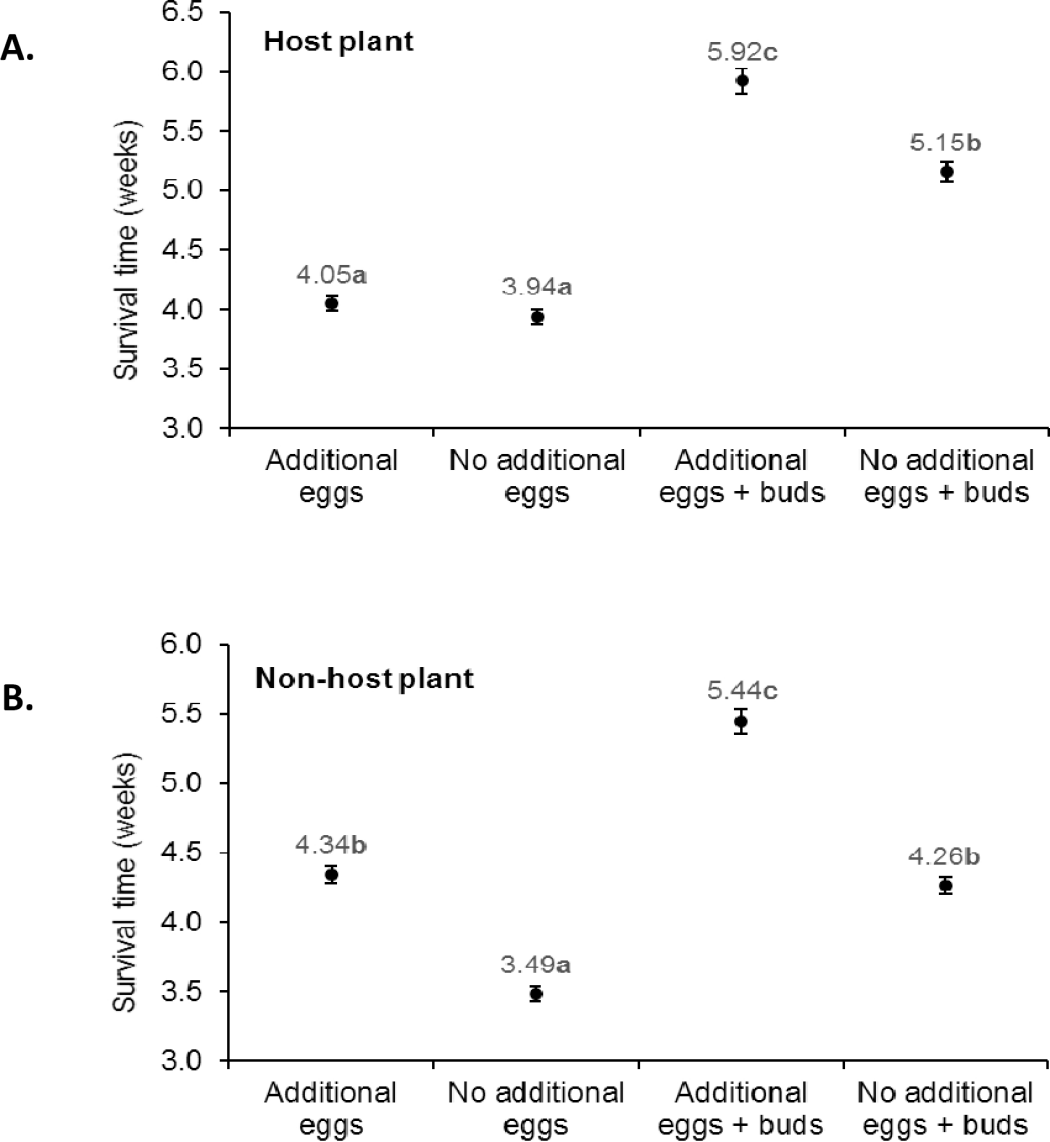
**Nymph survival time for core brood that hatched from egg masses collected on (A) host plants or (B) non-host plants**. Core brood were assigned to one of four treatments, with or without additional eggs and with or without flower buds as food. Points labeled with identical letters did not differ significantly (Weibull analysis, P>0.05). Vertical bars indicate 95% confidence internal.

A total of 26134 nymphs (12943 from egg masses collected from host plants, 13191 from egg masses on non-host plants) from 120 egg masses were monitored on a weekly basis over a 6-week period, after which 1832 individuals (1667 from egg masses collected from host plants, 165 nymphs from egg masses from non-host plants) were censored for analysis. Food availability, as additional eggs with or without flower buds, significantly influenced mean survival time of nymphs from egg masses that originated from the host plant (Weibull χ^2^ = 1648; d.f. = 3; P<0.001). Survival of nymphs from host plant egg masses was not affected by the presence of additional eggs (Fig 4A), whereas when flower buds were available the presence of additional eggs resulted in a significantly increased survival time compared to nymphs that had access to flower buds in the absence of additional eggs. The role of additional eggs and flower buds on nymphal survival was also significant for nymphs from egg masses collected on non-host plants (Weibull χ^2^ = 2515; d.f. = 3; P<0.001). In this case survival was significantly extended in the presence of additional eggs as a food source, when flower buds were both present and absent (Fig 4B).

### Do nymphs avoid sibling cannibalism of additional eggs if non-siblings are available?

The average (±SE) number of core brood eggs in experimental egg masses was 249.4 ± 15.7. The average number of additional eggs was 93.1 ± 7.5, of which half were removed and replaced with equal proportions of additional eggs from a different mother and core brood eggs from a different mother (together averaging 86.2 ± 5.8 eggs/mass). An average of 241.6 ± 15.7 nymphs emerged from the core brood eggs and 7.8 ± 2.1 eggs/mass (proportion 0.031) did not emerge. All the additional eggs offered were immediately consumed by nymphs with no differences in the consumption of any of the eggs from their own mother or a different mother (both core brood and additional eggs). None of these eggs survived or produced progeny. The survival of nymphs at two weeks post-eclosion was 100% and all had molted to the second instar, at which time they migrate in the field.

## Discussion

In the present study we examined a series of hypotheses related to maternal defense, material provisioning and oviposition site selection to elucidate the strategies employed by *R*. *insignis* to improve offspring survival before and after egg hatch. To examine the maternal defense hypothesis, females were removed from egg masses or remained with their core brood. Natural enemies markedly reduced the survival of eggs that were not protected by their mother. Females were extremely effective in defending progeny eggs from predatory ants, but less effective against parasitoid wasps. Parasitoids were often observed attempting to oviposit in eggs on the periphery of egg masses and despite the females frequent defensive kicks, almost a quarter of the core brood were parasitized in female guarded egg masses. These findings are consistent with those of others on the benefits of maternal guarding in sub-social bugs in which the eggs are attacked by predators [28–30], whereas effective protection against parasitoids is rare.

Removal of the additional eggs (when testing the provisioning hypothesis) did not result in a higher prevalence of parasitism of core brood eggs. It appears that in *R*. *insignis*, additional eggs are not deposited with the intention of providing core brood eggs with physical protection from the attack of natural enemies. Indeed, the asynchronous oviposition of *R*. *insignis* means that additional eggs are only present a few days prior to eclosion of nymphs so that any physical protection provided by additional eggs would be transient at best. However, laboratory rearing of additional eggs revealed that despite their brief period of availability, additional eggs of *R*. *insignis* were more heavily parasitized than core brood eggs, probably reflecting their exposed location at one extreme of the egg mass, and possibly a reduced degree of maternal protection from parasitoids given that these eggs were destined to be consumed by offspring. In contrast, the protection of core brood in other insects may involve placing trophic eggs at the periphery of eggs masses to act as a barrier to predators [31, 32], or by vertical stacking of eggs to protect offspring in the lowest layer, as observed in a bruchinid beetle [33] and probably in a burrowing bug (Cydnidae) [34]. The most valuable offspring may also be protected from natural enemies by varying maternal investment in eggs according to the risk of predation within each egg mass. Under such a scenario larger eggs are laid near the center of the egg mass where they are protected against predation by ants, whereas smaller eggs are laid at the edges of the egg mass where they are exposed to increased predation [28, 35].

To examine the maternal provisioning hypothesis additional eggs were removed or remained next to core brood eggs in the presence of their mother. Removal of the additional eggs resulted in an almost four-fold increase in the median prevalence of cannibalized eggs; an effect also observed in the maternal defense experiment in which females were removed prior to the oviposition of additional eggs. This tendency has also been observed in other species when trophic eggs were removed [16]. As additional eggs appear to provide core brood with nutritional resources during the early instars, failure to consume additional eggs is likely to be costly to nymphs as weight gain, developmental rate and survival are markedly increased in species in which this role is fulfilled by trophic eggs [32, 36, 37].

The functional significance of asynchronous oviposition and hatching was confirmed by comparison of the survival of nymphs provided or not with additional eggs and host plant food (flower buds). Provisioning of additional eggs improved survival of nymphs from host and non-host plants when flower buds were also available, indicating that flower buds alone cannot substitute for the nutritional benefits provided by additional eggs in *R*. *insignis*.

Laboratory rearing revealed that additional eggs were viable and hatched to produce nymphs that developed into adults, so that these eggs were likely to have been as costly to produce as core brood eggs. It is therefore clear that *R*. *insignis* adopts an ovipositional strategy based on marked temporal asynchrony in egg laying so that additional eggs are, in fact, marginal offspring produced to meet the nutritional needs of the core brood. As others have noted, the functional importance of eggs that do not have a clear trophic egg phenotype that favors their consumption by offspring (e.g., small size, fragile chorion, non-viable), can be that of nutrient provisioning when maternal behavior is modified to facilitate offspring feeding [18]. If a mother is not capable of selective fertilization or the production of a distinct trophic egg phenotype, she still may be capable of influencing which offspring are eaten by manipulating hatching asynchrony [38]. Indeed, the lacewing *Chrysoperla rufilabris* manipulates hatching period to favor greater heterogeneity in hatching times, facilitating higher levels of sibling cannibalism when food resources are scare [39], whereas hatching asynchrony is maternally manipulated to reduce cannibalism among sibling nymphs in a burrowing bug [40], or to favor survival of the last produced offspring in a predatory mite [41]. Alternatively, some species of bugs continue to produce trophic eggs after egg laying has ceased [42], or even after offspring have hatched resulting in increased nymphal survival [37].

In the choice experiment involving *R*. *insignis*, both non-sibling additional eggs and yellow-pink stage core brood eggs originating from other mothers were consumed rapidly following eclosion of nymphs, indicating that nymphs did not avoid consumption of marginal siblings (i.e., the additional eggs laid by their mother) immediately after hatching. This is unsurprising as the only eggs in the yellow stage of development encountered by newly hatched nymphs in nature are those provided by their mother. Under such circumstances there would be no benefit to be able to discriminate between siblings and non-siblings when feeding on additional eggs.

The benefits of the frequent use of non-host plants for oviposition were initially unclear given that *S*. *schreberi* is the only food plant of *R*. *insignis* in this region and migration from non-host plants in search of *S*. *schreberi* trees is likely to entail energetic costs and predation risks to migrating nymphs. Nonetheless, it became clear that oviposition site related differences in parasitism resulted in approximately one third more offspring for females that oviposited on non-host vegetation compared to those that oviposited on the host tree. This was because egg masses laid on the host tree were more likely to be discovered by parasitoids, although having been discovered, the prevalence of parasitism in egg masses was similar between both habitats. This probably reflects differences in the apparency of egg masses in each type of habitat [43]. Egg masses laid on host trees were high (~2.5 m) above the ground and not surrounded by plants other than the leaves of the host tree, whereas egg masses laid on non-host vegetation were nearer to the ground (0.9 m) and almost invariably in close association with other plant species of similar height. As such parasitoids searching non-host vegetation may have experienced difficulty in locating egg masses due to the complexity of infochemical cues present in the ground vegetation community [44].

Female *R*. *insignis* adjusted the number of additional eggs according to oviposition site, presumably in response to the increased energetic costs and risk of starvation experienced by nymphs during migration from non-host vegetation, in line with the provisioning hypothesis. Each additional egg supplied an average of 5.0 nymphs with nutritional resources for clutches laid on the host tree compared to an average of 2.7 nymphs per additional egg for clutches laid on non-host vegetation. A similar response occurred in a cydnid bug in which an increase in the ratio of trophic eggs to core brood eggs was observed in response to a decrease in the quality of dietary resources [45]. The nutritional importance of the additional eggs of *R*. *insignis* was further underlined by the early migration response of nymphs from egg masses from which the additional eggs had been removed, presumably motivated by their imminent starvation.

Insects often face a tradeoff during oviposition site decision making due to site specific differences in the abundance of natural enemies for those insects that choose to oviposit on non-standard host plants [46, 47]. The downside of selecting non-standard hosts can include fitness costs of reduced food quality [48], toxic plant defenses [49], and temporal variation in the abundance and diversity of natural enemies [50] and their search behavior [51]. Our findings highlight two questions. First, what are the costs to the offspring in terms of depletion of energy reserves and mortality risks during migration from ground vegetation to the host tree? Several of our attempts at estimating the survival rates of migrating nymphs using video cameras and direct observation failed due to the complexity of the habitat. However, energetic costs might be estimated indirectly by comparing the growth or body weight of nymphs undergoing migration with non-migrating conspecifics on host plants, following the approaches taken in studies on the functional role of trophic eggs in sub-social bugs [32, 36]. Second, higher provisioning by females on non-host vegetation might indicate that oviposition site selection is mediated by female quality. In such a case, large females with high levels of resources would select non-host plants for oviposition with lower risks of offspring losses to parasitoids traded off against costs for offspring migration, whereas smaller or resource deprived females would select a host tree oviposition site with higher levels of parasitism traded off against ready access to food resources for the offspring. The validity of these hypotheses depends crucially on the risk of mortality during migration to the host tree. If larger cohorts have a lower *per capita* risk of predation during migration then oviposition on non-host plants may be costly to small females that tend to produce smaller clutches and beneficial to larger females that lay large clutches. However, we found no evidence that larger or heavier females selected non-host plants over host plants for oviposition so that factors that modulate the allocation of maternal resources to reproduction and maternal provisioning in *R*. *insignis* remain the subject of future studies. We conclude that *R*. *insignis* females present a remarkable combination of maternal defense against natural enemies, provisioning of additional eggs and oviposition site selection as strategies to improve offspring survival in the egg and nymph stages.

## Supporting information

S1 File Original data from all studies. (XLSX)

## References

[1] Singer MC. The definition and measurement of oviposition preference In: Miller J, Miller TA, editors. Insect–plant relationships. New York: Springer-Verlag; 1986 pp. 65–93.

[2] Bernays EA, Chapman RF. Host-plant selection by phytophagous insects New York: Chapman Hall; 1994.

[3] Ohsaki N, Sato Y. Food plant choice of *Pieris* butterflies as a trade-off between parasitoid avoidance and quality of plants. Ecology. 1994; 75: 59–68.

[4] Conti E, Roversi PF, Bin F. Morphofunctional adaptations of parasitoids attacking concealed eggs of two arboreal mirids in Italy. J Hymenopt Res. 2000; 9: 385–394.

[5] Udayagiri S, Welter SC. Escape of *Lygus hesperus* (Heteroptera: Miridae) eggs from parasitism by *Anaphes iole* (Hymenoptera: Mymaridae) in strawberries: plant structure effects. Biol Control. 2000; 17: 234–242.

[6] Meiners T, Randlkofer B, Obermaier E. Oviposition at low temperatures—late season negatively affects the leaf beetle *Galeruca tanaceti* (Coleoptera: Galerucinae) but not its specialised egg parasitoid *Oomyzus galerucivorus* (Hymenoptera: Eulophidae). Eur J Entomol. 2006; 103: 765–770.

[7] Fox LR, Eisenbach J. Contrary choices: possible exploitation of enemy-free space by herbivorous insects in cultivates vs. wild crucifers. Oecologia. 1992; 89: 574–579. doi: 10.1007/BF00317166 28311890 10.1007/BF00317166

[8] Murphy SM. Enemy-free space maintains swallowtail butterfly host shift. Proc Nat Acad Sci USA. 2004; 101: 18048–18052. doi: 10.1073/pnas.0406490102 15601773 10.1073/pnas.0406490102 PMC539780

[9] Moon DC, Stiling P. Trade-off in oviposition strategy: choosing poor quality host plants reduces mortality from natural enemies for a salt marsh planthopper. Ecol Entomol. 2006; 31: 236–241.

[10] Perfecto I, Vet LEM. Effect of a nonhost plant on the location behavior of two parasitoids: the tritrophic system of *Cotesia* spp. (Hymenoptera: Braconidae), *Pieris rapae* (Lepidoptera: Pieridae), and *Brassica oleraceae*. Env Entomol. 2003; 32: 163–174.

[11] Gols R, Bukovinszky T, Hemerik L, Harvey JA, Lenteren JC, Vet LEM. Reduced foraging efficiency of a parasitoid under habitat complexity: implications for population stability and species coexistence. J Anim Ecol. 2005; 74: 1059–1068.

[12] Tallamy DW, Brown WP. Semelparity and the evolution of maternal care in insects. Anim Behav. 1999; 57: 727–730. doi: 10.1006/anbe.1998.1008 10196065 10.1006/anbe.1998.1008

[13] Cocroft RB. Antipredator defense as a limited resource: unequal predation risk in broods of an insect with maternal care. Behav Ecol. 2002; 13: 125–133.

[14] Scott MP. The ecology and behavior of burying beetles. Ann Rev Entomol. 1998; 43: 595–618. 15012399 10.1146/annurev.ento.43.1.595

[15] Filippi L, Hironaka M, Nomakuchi S. Risk-sensitive decisions during nesting may increase maternal provisioning capacity in the subsocial shield bug *Parastrachia japonensis*. Ecol Entomol. 2002; 27: 152–162.

[16] Crespi BJ. Cannibalism and trophic eggs in subsocial and eusocial insects In: Elgar B, Crespi BJ, editors. Cannibalism: ecology and evolution among diverse taxa. Oxford, UK: Oxford University Press; 1992 pp. 176–213.

[17] Perry JC, Roitberg BD. Ladybird mothers mitigate offspring starvation risk by laying trophic eggs. Behav Ecol Sociobiol. 2005; 58: 578–586.

[18] Perry JC, Roitberg BD. Trophic egg laying: hypotheses and tests. Oikos. 2006; 112: 706–714.

[19] Mock DW, Forbes LS. The evolution of parental optimism. Trends Ecol Evol. 1995; 10: 130–134. 21236982 10.1016/s0169-5347(00)89014-x

[20] Lamey TC, Evans RM, Hunt JD. Insurance reproductive value and facultative brood reduction. Oikos. 1996; 77: 285–290.

[21] Alexander RD. The evolution of social behavior. Ann Rev Ecol Syst. 1974; 5: 325–383.

[22] Polis GA. The evolution and dynamics of intraspecific predation. Ann Rev Ecol Syst. 1981; 12: 225–251.

[23] Kormilev NA. Sobre los géneros *Vulsirea* Spinola (1837), *Ramosiana* Kormilev (1950) y *Adoxoplatys* Breddin (1903), con la descripción de tres especies nuevas (Hemipt. Pentat.). Rev Soc Entomol Arg. 1951; 15: 83–95.

[24] López M, Cervantes L. Life histories of *Ramosiana insignis* (Blanchard) and *Vulsirea violacea* (F.)(Hemiptera-Heteroptera: Pentatomidae), with descriptions of immature stages. Proc Entomol Soc Wash. 2010; 112: 81–96.

[25] López-Ortega M, Pérez-Rodríguez P, Rojas JC, Hernández RM, López-Mata L, Rico-Gray V. Host use and resource sharing by fruit/seed-infesting insects on *Schoepfia schreberi* (Olacaceae). Env Entomol. 2013; 42: 231–239. 23575012 10.1603/EN12284

[26] Numerical Algorithms Group. Generalised linear interactive modelling GLIM 4. Oxford, UK: Clarendon Press; 1993.

[27] Crawley MJ. Glim for ecologists Oxford, UK: Blackwell; 1993.

[28] Mappes J, Mappes T, Lappalainen T. Unequal maternal investment in offspring quality in relation to predation risk. Evol Ecol. 1997; 11: 237–243.

[29] Nakahira T, Kudo S. Maternal care in the burrower bug *Adomerus triguttulus*: defensive behavior. J Insect Behav. 2008; 21: 306–316.

[30] Williams L III, Coscarón MC, Dellapé PM, Roane TM. The shield-backed bug, *Pachycoris stallii*: description of immature stages, effect of maternal care on nymphs, and notes on life history. J Ins Sci. 2005; 5: 29. 10.1093/jis/5.1.29 PMC1615236 17119611

[31] Nakahira T. Trophic egg production in the subsocial burrower bug *Admerus triguttulus*. Naturwissenschaften. 1994; 81: 413–414.

[32] Kudo S, Nakahira T. Effects of trophic eggs on offspring performance and rivalry in a sub-social bug. Oikos. 2004; 107: 28–35.

[33] Deas JB, Hunter MS. Mothers modify eggs into shields to protect offspring from parasitism. Proc R Soc B. 2011; 279: 847–853. doi: 10.1098/rspb.2011.1585 21920977 10.1098/rspb.2011.1585 PMC3259937

[34] Kudo S, Nakahira T, Saito Y. Morphology of trophic eggs and ovarian dynamics in the subsocial bug *Adomerus triguttulus* (Heteroptera: Cydnidae). Can J Zool. 2006; 84: 723–728.

[35] Kudo SI. Intraclutch egg‐size variation in acanthosomatid bugs: adaptive allocation of maternal investment? Oikos. 2001; 92: 208–214.

[36] Hironaka M, Nomajuchi S, Iwakuma S, Filippi L. Trophic egg production in a subsocial bug, *Parastrichia japonensis* Scott (Heteroptera: Parastrichiidae) and its functional value. Ethology. 2005; 111: 1089–1102.

[37] Filippi L, Baba N, Inadomi K, Yanagi T, Hironaka M, Nomakuchi S. Pre- and post-hatch trophic egg production in the subsocial burrower bug, *Canthophorus niveimarginatus* (Heteroptera: Cydnidae). Naturwissenschaften. 2009; 96: 201–211. doi: 10.1007/s00114-008-0463-z 18846358 10.1007/s00114-008-0463-z

[38] Godfray HCJ. The evolution of clutch size in invertebrates. Oxford Surv Evol Biol. 1987; 4: 117–154.

[39] Fréchette B, Corderre D. Oviposition strategy of the green lacewing *Chrysoperla rufilabris* (Neuroptera: Chrysopidae) in response to extraguild prey availability. Eur J Entomol. 2000; 97: 507–510.

[40] Mukai H, Hironaka M, Tojo S, Nomakuchi S. Maternal hatching synchronization in a subsocial burrower bug mitigates the risk of future sibling cannibalism. Ecol Evol. 2018; doi: 10.1002/ece3.3894 10.1002/ece3.3894 PMC5869296 29607032

[41] Schausberger P, Hoffman D. Maternal manipulation of hatching asynchrony limits sibling cannibalism in the predatory mite *Phytoseiulus persimilis*. J Anim Ecol. 2008; 77: 1109–1114. doi: 10.1111/j.1365-2656.2008.01440.x 18624737 10.1111/j.1365-2656.2008.01440.x

[42] Kudo S, Nakahira T. Trophic egg production in a subsocial bug: adaptive plasticity in response to resource conditions. Oikos. 2005; 111: 459–464.

[43] Obermaier E, Heisswolf A, Randlkofer B, Meiners T. Enemies in low places—insects avoid winter mortality and egg parasitism by modulating oviposition height. Bull Entomol Res. 2006 96: 337–343. 16923200

[44] Randlkofer B, Obermaier E, Meiners T. Mother's choice of the oviposition site: balancing risk of egg parasitism and need of food supply for the progeny with an infochemical shelter?. Chemoecology. 2007; 17: 177–186.

[45] Kudo S, Nakahira T, Saito Y. Morphology of trophic eggs and ovarian dynamics in the subsocial bug *Adomerus triguttulus* (Heteroptera: Cydnidae). Can J Zool. 2006; 84: 723–728.

[46] Eitam A, Blaustein L. Oviposition habitat selection by mosquitoes in response to predator (*Notonecta maculata*) density. Physiol Entomol 2004; 29: 188–191.

[47] Wiklund C, Friburg M. Enemy free space and habitat specific host specialization in a butterfly. Oecologia. 2008; 157: 287–294. doi: 10.1007/s00442-008-1077-z 18607636 10.1007/s00442-008-1077-z

[48] Singer MS, Rodrigues D, Stireman III JO, Carriere Y. Roles of food quality and enemy free space in host use by a generalist insect herbivore. Ecology. 2004; 85: 2747–2753.

[49] Heard SB, Stireman JO III, Nason JD, Cox GH, Kolacz CR, Brown JB. On the elusiveness of enemy free space: spatial, temporal and host-plant related variation in parasitoid attack rates on three gallmakers of goldenrods. Oecologia. 2006; 150: 421–434. doi: 10.1007/s00442-006-0529-6 10.1007/s00442-006-0529-6 16944244

[50] Kessler A, Baldwin IT. *Manduca quinquemaculata*'s optimization of intra-plant oviposition to predation, food quality and thermal constraints. Ecology. 2002; 83: 2346–2354.

[51] Gratton C, Welter SC. Does "enemy-free space" exist? Experimental host shifts of an herbivorous fly. Ecology. 1999; 80: 773–785.

